# Transcriptome Profiling Identifies Ribosome Biogenesis as a Target of Alcohol Teratogenicity and Vulnerability during Early Embryogenesis

**DOI:** 10.1371/journal.pone.0169351

**Published:** 2017-01-03

**Authors:** Mark E. Berres, Ana Garic, George R. Flentke, Susan M. Smith

**Affiliations:** Department of Nutritional Sciences, University of Wisconsin-Madison, Madison, Wisconsin, United States of America; Universite de Liege, BELGIUM

## Abstract

Fetal alcohol spectrum disorder (FASD) is a leading cause of neurodevelopmental disability. Individuals with FASD may exhibit a characteristic facial appearance that has diagnostic utility. The mechanism by which alcohol disrupts craniofacial development is incompletely understood, as are the genetic factors that can modify individual alcohol vulnerability. Using an established avian model, we characterized the cranial transcriptome in response to alcohol to inform the mechanism underlying these cells’ vulnerability. *Gallus gallus* embryos having 3–6 somites were exposed to 52 mM alcohol and the cranial transcriptomes were sequenced thereafter. A total of 3422 genes had significantly differential expression. The KEGG pathways with the greatest enrichment of differentially expressed gene clusters were Ribosome (*P* = 1.2 x 10^−17^, 67 genes), Oxidative Phosphorylation (*P* = 4.8 x 10^−12^, 60 genes), RNA Polymerase (*P* = 2.2 x 10^−3^, 15 genes) and Spliceosome (*P* = 2.6 x 10^−2^, 39 genes). The preponderance of transcripts in these pathways were repressed in response to alcohol. These same gene clusters also had the greatest altered representation in our previous comparison of neural crest populations having differential vulnerability to alcohol-induced apoptosis. Comparison of differentially expressed genes in alcohol-exposed (3422) and untreated, alcohol-vulnerable (1201) transcriptomes identified 525 overlapping genes of which 257 have the same direction of transcriptional change. These included 36 ribosomal, 25 oxidative phosphorylation and 7 spliceosome genes. Using a functional approach in zebrafish, partial knockdown of ribosomal proteins *zrpl11*, z*rpl5a*, and *zrps3a* individually heightened vulnerability to alcohol-induced craniofacial deficits and increased apoptosis. In humans, haploinsufficiency of several of the identified ribosomal proteins are causative in craniofacial dysmorphologies such as Treacher Collins Syndrome and Diamond-Blackfan Anemia. This work suggests ribosome biogenesis may be a novel target mediating alcohol’s damage to developing neural crest. Our findings are consistent with observations that gene-environment interactions contribute to vulnerability in FASD.

## Introduction

Fetal alcohol spectrum disorder (FASD) is a leading cause of permanent neurodevelopmental disability [1]. Recent estimates for the U.S. suggest it affects 2.8% to 4.6% of school-aged children [2] with higher rates in populations with substantial alcohol abuse [3]. Work in animal models shows that prenatal alcohol exposure (PAE) disrupts neurodevelopmental events including neuronal survival, proliferation, migration, and synaptogenesis [4]. Alcohol affects cellular activity through its interactions with target proteins, wherein alcohol displaces water from select binding sites to alter protein structure and activity [5]. Previously we and others have reported that alcohol causes the apoptotic death of an early neuroprogenitor population called the neural crest [6–8]. Disruptions in neural crest development contribute to a characteristic craniofacial dysmorphology that can be diagnostic for some individuals who exhibit FASD morphological characteristics [9,10]. Using an established chick embryo model of alcohol exposure, we showed that alcohol causes craniofacial structural changes consistent with those of the human disorder [6,9]. We further showed that alcohol causes apoptosis within neural crest progenitors because it elicits the phosphoinositide-mediated efflux of calcium from intracellular stores [11]. The subsequent activation of CaMKII phosphorylates and destabilizes transcriptionally active β-catenin, removing essential trophic support from these cells [12,13].

Vulnerability to FASD varies greatly and can be influenced by pattern and dose of alcohol consumption. Gene-environment interactions also shape alcohol vulnerability. Early studies documented that allelic differences in maternal alcohol metabolism affect peak blood alcohol concentrations and clearance rates, and thereby FASD risk [14]. Genes and signaling pathways that govern cell survival and development also make significant contributions. For example, loss-of-function mutations within the *sonic hedgehog* (shh) signaling pathway heighten the vulnerability of developing craniofacial and brain cell populations to alcohol [15,16], as do genes encoding *Vangl2*, *MARS*, and *PDGFRA* [8,17]. Allelism in *PDGFRA* is also linked to alcohol vulnerability in human FASD [8]. Additional candidates at the pathway level have been revealed though systems-level comparisons of genetically-related strains having differential vulnerability to alcohol-induced teratogenicity. For the headfold-stage mouse embryo, a developmental stage similar to that studied herein, comparisons of strains having differential alcohol sensitivity identified multiple KEGG clusters having altered representation in response to alcohol including methylation, chromatin organization, pentose phosphate pathway, glycolysis / gluconeogenesis, ribosome, mRNA splicing, and proteasome [18,19].

We recently performed a similar analysis upon experimentally-naïve, cranial headfolds isolated from genetically-related chick strains that have a well-characterized differential vulnerability to alcohol-induced apoptosis and calcium mobilization [20]. This transcriptome-level analysis relied on high-throughput sequencing, due to the dynamic annotation of the chick genome, and revealed significantly altered representation of gene families corresponding to ribosome biogenesis, oxidative phosphorylation, and spliceosomal signaling pathways, as well as candidate genes that directly mapped to the previously documented, calcium / β-catenin apoptotic pathway. That several of these KEGG sets (ribosome, splicing, proteasome) were conserved between chick and mouse suggested these pathways might also contribute to the alcohol responses of other vertebrate species including human. Here, we extend that work and characterize the transcriptome of these same headfold populations in direct response to alcohol challenge, hypothesizing that this unbiased approach would reveal novel mechanistic insights into candidate pathways that could potentially mediate alcohol’s developmental toxicity. We further hypothesized that comparison of this gene set against the previously described transcriptome comparison of untreated headfolds might additionally reveal candidate genes that could potentially modify embryo vulnerability to alcohol. We find that gene clusters displaying the greatest response to alcohol challenge are in the same pathways having the greatest differential expression in the comparison of experimentally-naïve vulnerable and resistant cell populations. Haploinsufficiencies in one such gene cluster, ribosome, increase embryo vulnerability to alcohol-induced apoptosis and cranial dysmorphology. This transcriptome comparison approach offers novel insights into the mechanisms that mediate both alcohol’s neurotoxicity and genetic vulnerability to that cellular damage.

## Methods

### Embryo Generation and Ethanol Treatment

Fertile chicken eggs (*Gallus gallus*) from the commercial layer line Special Black were obtained from Sunnyside Egg Farm (Beaver Dam, WI). This line was derived approximately 10 generations ago by crossing Rhode Island Red females with Dekalb Black males. Embryos were incubated to approximately Hamburger-Hamilton stage 8- to stage 8 (3–4 somites, 27 hr incubation), at which time eggs were injected with 0.43 mmol USP grade ethanol or isotonic saline as described [21] and reincubated. Embryos experience a peak alcohol concentration of 50–60 mM ethanol for 90 min, then levels decline rapidly thereafter to baseline (9mM) [22]. The neural folds anterior to somite pair two were isolated six hours thereafter and flash-frozen. The preponderance of cranial neural folds comprises neuroprogenitors including neural crest, with minor contributions from mesendoderm, notochord, and the secondary heart fields.

### cDNA Preparation and Sequencing

RNA isolation and quality assurance, as well as cDNA synthesis, purification, and quality assurance, were performed exactly as described previously [20]. Paired-end reads (75 bp) were generated on an Illumina Genome Analyzer IIx (University of Wisconsin-Madison Biotechnology Center) with each sample occupying an independent lane on the same flow cell. We sequenced 2 biological replicates for each treatment (two alcohol-treated, two saline-treated). Each sample contained a pool of 23 individual neural folds and each treatment sequenced a total of 46 individual neural folds. Samples were identically-matched with respect to developmental stage and each contained 5 heads at stage 8+, 5 heads at stage 9-, 9 heads at stage 9, and 4 heads at stage 9+. This precise distribution of developmental stage is identical to that used in our previous characterization of candidate genes that modify alcohol vulnerability [20], and the careful control of staging enabled direct comparison of the sequence data sets from these two studies. We showed elsewhere that headfolds of embryos having 5–8 somites (stages 8+ through 9+) have equivalent alcohol responses with respect to apoptosis and facial outcome [21,22] and their cranial neural crest is poised at the onset of epithelial-mesenchymal transformation.

### Data Analysis

Analysis of sequencing data was performed as described previously [20], with minimal modification. In brief, RNA-Seq sequence reads were filtered through 4 trimming procedures that removed: (i) reads failing to meet or exceed Illumina’s default chastity threshold of 0.6 over the first 25 cycles; (ii) reads containing more than 2 contiguous or interspersed ambiguous nucleotides; (iii) reads with quality scores <20 (Phred scale; 1 error per 100 nucleotides); and (iv) reads that were shorter than the expected 75 bp. The RNA-Seq module in CLC Genomics Workbench 5.5 (CLC Bio, Cambridge, MD) was used to map the filtered paired-end reads to 17,108 genes (known or predicted) derived from assembly 4.0 of the *Gallus gallus* genome (Ensembl release 73; September 2013). Reads were mapped to the reference genome, individually requiring at least 90% of each read to exhibit 80% or greater alignment similarity. Only reads that mapped uniquely to the reference were included in subsequent analyses. Raw counts of read mappings were normalized to the reference genome using DESeq v1.10.1 [23] as described previously [20].

All *p*-values were adjusted with the Benjamin-Hochberg multiple testing correction to control the false discovery rate (FDR). Data were evaluated as mean normalized counts vs. dispersion, and as mean normalized counts vs. log2 fold change, plotting both empirical dispersion and fitted values for the former. Because DESeq is generally considered to use a conservative algorithm, genes were deemed differentially expressed if the adjusted level of significance was below 0.10. Genes identified as differentially expressed (DE) were submitted to DAVID [24,25] for GO term analysis. Using the Kyoto Encyclopedia of Genes and Genomes KEGG [26], pathway analysis was also performed in DAVID v6.7 using the above parameters.

Our previous comparison [20] of differential gene expression in the neural folds of experimentally-naïve alcohol-vulnerable (Hy-Line W98S) and alcohol-resistant (Hy-Line W98D) embryos used galgal3 release e70 (Ensembl, January 2013). Because gene annotation often improves with new database releases, we re-analyzed those data by mapping the same RNA-Seq reads to galgal4 release e73 (Ensembl, September 2013) as above, such that all data sets would be consistent with the revised genome assembly in the galgal4 release. We then determined the sets of differentially expressed genes in both datasets. An enrichment analysis for both GO terms and KEGG pathways was then performed on this set of differentially expressed genes as described above. Finally, we determined the intersection of differentially expressed genes in the vulnerable/responder sets and tested those genes for GO term and KEGG pathway enrichment. All data utilized herein from study [20] thus represent a reanalysis using the updated galgal4 release e73.

### Zebrafish Morpholino Study

Translation-blocking morpholinos were designed and purchased from Gene Tools (Philomath, OR). Sequences used in this study were (5’-TTTGCCGACTGCCATGTGAACAC-3’) for RPS3A (MO1-rps3a) [27], (5’-ACCCATTTTGTGATCGTTTGTTCTC-3’) for RPL5A (MO1-rpl5a) [27], and (5’-CTTCTTCTCGCTCTGGTCCGCCATG-3’) for RPL11 (MO1-rpl11) [27,28]. A standardized nonsense morpholino (5’-CCTCTTACCTCAGTTACAATTTATA-3’) was used as an irrelevant sequence control. Zebrafish (*Danio rerio*) eggs (outbred strain 5D, gift of R. Tanguay, Oregon State University) at the 1-cell or 2-cell stage were collected and sorted from an on-site population of breeders. Zebrafish studies were reviewed and approved by the UW-Madison IACUC under protocol #A005104. Approximately 5 nl of morpholino diluted in 1X Danieau solution was injected into the yolk with a beveled 20 μM inner-diameter bore micropipette (Origio A/S, Denmark) that was connected to an ASI MPPI-3 pulse injector (Eugene, OR). The concentration of each morpholino injected was 30 μM (*rps3a*), 600 μM (*rpl5a*), 0.0625 μM (*rpl11*), and 100 μM (control), and were defined empirically such that they did not cause appreciable apoptosis or facial dysmorphology, minimizing risks for off-target effects [29]. At 70% epiboly, chorionated embryos were exposed for 3 hrs to 241 mM ethanol (1.5%) in fish water, and this dose produces an alcohol concentration within the embryo of 86 mM (0.5%) [30]. This dosage and exposure window causes a calcium-dependent neural crest apoptosis and facial malformations in zebrafish embryos which parallel those of the chick model [30]. At four days post-fertilization (dpf), embryos injected with either *rps3a* or *rpl5a* were euthanized and stained with Alcian Blue following the protocol described in Carvan, et al., [31]. Injection into the embryo of *rpl11* morpholino at concentrations at greater than 0.25 μM were lethal or caused abnormal development at 24 hpf. When the concentration of *rpl11* morpholino was reduced to 0.0625 μM, embryos survived and were morphologically comparable with control embryos through 3 dpf, at which time they were processed in the same manner as RPS3A and RPL5A.

The DeadEnd Fluorometric TUNEL System (Promega, WI) was used to visualize fragmented DNA in apoptotic cells. Zebrafish embryos were injected with *rps3a*, *rpl5a*, or *rpl11* morpholino as described above. Embryos were enzymatically dechorionated with Pronase [32] at 4hpf, and treated at 75% epiboly with 42 mM alcohol for three hours. The alcohol was then washed away and the embryos were incubated an additional 2hr without alcohol and then were fixed at 12hpf (~4–6 somites) and stained for TUNEL at 12hpf as previously described [30]. Enumeration of TUNEL^+^ cells in 10–12 embryos per treatment was analyzed using a one-way analysis of variance followed by pairwise multiple comparisons using the Holm-Sidak method (SigmaPlot, San Jose CA).

## Results

### Differential Gene Expression in Response to Alcohol

Whole transcriptome sequencing of cranial neural folds treated with alcohol or isotonic saline yielded 92.2 and 84.2 million reads, respectively, after trimming. The cumulative distribution of read alignments across transcript targets was nearly identical in each dataset, with 80.84% and 81.90% mappable reads for alcohol and saline, respectively. Of those sequences that mapped to an annotated region in assembly 4.0 of the *Gallus gallus* genome (Ensembl release 73; September 2013), 78.7% and 78.5% mapped uniquely and exhibited an average mismatch per base of 0.53%. The number of reads mapping to two or more sites averaged 2.17% and 3.48%, respectively. Because technical variation is generally accepted to be minimal in RNA-Seq platforms [33], the magnitude of transcript count dispersion derives primarily from biological variation. A plot of normalized expression vs. magnitude of dispersion in saline and alcohol treatments showed that dispersion decreased as read counts increased (S1A Fig). However, many values below the fitted values in the dispersion plot may underestimate the true biological dispersion. To be conservative, the DESeq algorithm moves all empirical dispersion values below the fitted estimate to that estimate. All the other empirical values above the fitted line remain, even if some of these are over-estimates of dispersion [23]. Thus, more dispersion between read counts in saline and alcohol treatments, due to true biological variation, is required for statistical significance. We found 3,422 genes were differentially expressed (BH corrected FDR at P < 0.10; S1 Table) between the transcriptomes of neural folds treated with alcohol or isotonic saline; the preponderance (80.6%, 2849 genes) were below the FDR cut-off of <0.05. The plot of normalized expression versus log_2_ fold-change of transcript abundance (S1B Fig) shows more down-regulated (1,924) than up-regulated (1,498) transcripts in cells treated with alcohol.

GO term analysis of the 3,422 differentially expressed transcripts revealed that the functional designations of genes with the most enriched transcript representation were Biological Process (723 genes), Cellular Component (1182 genes), and Molecular Function (1780 genes) (Table 1). The five most significant enrichments of GO terms within each category include genes associated with ribosome biogenesis, oxidative phosphorylation processes, and protein kinase activities. Importantly, these significant enrichments distributed among the three functional designations were strikingly similar to those previously identified as having altered expression in the experimentally-naïve alcohol-vulnerable cranial neural folds that were analyzed in the absence of alcohol exposure [20]. The gene distribution within these GO subcategories is presented in S2 Fig.

**Table 1:** Gene Ontogeny Term Enrichments for Differentially Expressed Genes in Alcohol-Exposed Cranial Neural Folds.

	# Genes	% of Total	BH-Corrected *P*-Value
**Biological Processes (723 genes)**			
Translation	85	2.9	4.70 x 10^−15^
Phosphorus metabolic process	149	5.2	4.90 x 10^−05^
Phosphate metabolic process	149	5.2	4.90 x 10^−05^
Phosphorylation	127	4.4	2.00 x 10^−04^
Generation of precursor metabolites and energy	48	1.7	6.50 x 10^−04^
**Cellular Component (1182 genes)**			
Ribosome	67	2.3	1.10 x 10^−20^
Ribonucleoprotein complex	87	3.0	2.30 x 10^−18^
Mitochondrion	105	3.6	2.20 x 10^−109^
Mitochondrial part	58	2.0	1.90 x 10^−06^
Mitochondrial membrane	46	1.6	1.50 x 10^−05^
**Molecular Function (1780 genes)**			
Structural constituent of the ribosome	62	2.1	1.50 x 10^−19^
Protein serine/threonine kinase activity	71	2.5	2.80 x 10^−03^
Protein kinase activity	97	3.4	3.40 x 10^−02^
RNA binding	62	2.1	3.80 x 10^−02^
Nucleotide binding	290	10.0	3.10 x 10^−02^

The most statistically significant pathway identified by KEGG enrichment was Ribosome (#03010; *P* = 1.2 x 10^−17^) with 53 (see below) genes having altered expression. Other significantly enriched pathways in response to alcohol exposure included oxidative phosphorylation (#00190; 60 genes, *P* = 4.8 x 10^−12^), RNA polymerase (#03020; 15 genes, *P* = 2.2 x 10^−03^) and spliceosome (#03040; 39 genes, *P* = 2.6 x 10^−02^). These gene lists are presented in Table 2.

**Table 2:** KEGG Pathway Enrichments in Alcohol-Exposed Cranial Neural Folds.

KEGG Pathway	# Genes*	BH-CorrectedSignificance	Gene Name
Ribosome	53 (67)	1.2 x 10^−17^	RPL3, RPL4, RPL5, RPL6, RPL7, RPL7A, RPL8, PRL9**, RPL10A, RPL10L**, RPL11/TCEB3**, RPL12, RPL13, RPL14, RPL15, RPL17L, RPL18A, RPL19, RPL21**, RPL22, RPL22L1**, RPL23, RPL23A, RPL24, RPL26, RPL27, RPL27A, RPL29**, RPL30, RPL31, RPL32**, RPL35, RPL35A, RPL36, RPL37, RPL37A, RPL38, RPL39, RPLP0, RPLP1, RPLP2, RPS2**, RPS3, RPS3A, RPS4, RPS6, RPS6KA3**, RPS6KA6**, RPS6KB2**, RPS8, RPS10**, RPS11, RPS12, RPS13, RPS14, RPS15, RPS15A, RPS16**, RPS17**, RPS19BP1**, RPS20, RPS23**, RPS24, RPS25, RPS26**, RPS27A, RPS28, RPS29, RPSA
Oxidative Phosphorylation	60	4.8 x 10^−12^	SDHB, NDUFA2, UQCR11, ATP5G1, NDUFA8, ATP5F1, ATP5A1W, NDUFB6, ATP6V1G3, UQCRC2, NDUF55, PPA1, NDUFB10, NDUFB8, COX4I1, UQCRC1, NDUFAB1, ATP5C1, ATP6V1G1, COX6A1, COX15, SDHD, ATP5H, UQCR10, NDUFS3, NDUFB3, NDUFA1, NDUFB5, ATP6V0C, ATP5G3, ATXN3, NDUFS6, COX8A, COX5A, NDUFV2, ATP6V1A, RBF, ATP5J, COX7A2, ATP5O**, NDUFB9, UQCRH, NDUFS8, MT-CYB, MT-ND5, MT-ND4, MT-ND3, MT-CO3. ATP6, gga-mir-3527, MT-CO1, MT-ND2, MT-ND1, ATP6V0E2, NDUFA11**, ND4L, ATP5B
RNA Polymerase	15	2.2 x 10^−03^	POLR2C, RASA4**, POLR2D, POLR2E, POLR3A, POLR2L, POLR1D, POLR2H, POLR3F; POLR1C, POLR3H, RPB6, POLR2I, POLR1A, POLR3K
Spliceosome	39	2.6 x 10^−02^	SNRPE, HNRNPM, LSM7, SNRNP40, ZMAT2, SF3A3, BCAS2, SNRPC, LSM4, BUD31, PPIH, CCDC12, SNRPD3, SNRPA1, ALYREF, SNRPB, EIF4A3, PRPF4, NAA38, HNRNPA3, AQR, PRPF38A, MAGOH, SNRPF, HSP70, SNRPD1, NHP2L1, PPIE, SYF2, SRSF7, FAM136A, SNRNP27, PRPF19, PUF60, THOC1, U2AF1, SF3B14, CWC15, THOC3
Cardiac MuscleContraction	20	2.2 x 10^−01^	TNNT2, UQCR11, TNNC1, UQCRC2, ATP1B3, COX4I1, UQCRC1, COX6A1, LOC771947**, UQCR10, CACNA2D1, ACTC1, ACTA1, COX8A, COX5A, COX7A2, UQCRH, MT-CYB, MT-CO3, gga-mir-3527, MT-CO1

* The number of genes reflects e73 annotation (Sept 2013), but gene names may have since been assigned in e78 (Dec 2014). Thus, the number of gene names may exceed the total number of genes.

** For genes that were left blank or listed as Novel in e73, we used the most recent annotation, e78.

### Comparison of Alcohol-Responsive and Alcohol-Vulnerable Gene Sets

Three out of four of the KEGG gene pathways with significantly enriched gene representation 6 hr post-alcohol challenge (ribosome, oxidative phosphorylation, spliceosome) were the same three KEGG pathways that had significantly altered representation in our previous comparison of alcohol-vulnerable and alcohol-resistant cranial neural folds, which were analyzed in the absence of alcohol exposure [20]. We hypothesized that the intersection of these two gene sets could represent candidate genes for modulating alcohol vulnerability. The original analysis of experimentally-naïve, alcohol vulnerable/resistant neuroprogenitors utilized the Galgal3 e70 (January 2013) genome assembly, and to facilitate the present comparison we first remapped those data onto Galgal4 e73 (September 2013). Afterwards, the number of differentially expressed transcripts increased from 363 [20] to 1201 (S2 Table), and 74.5% (895 genes) of these were below the FDR cut-off of 0.05. Of the original 363 differentially expressed genes, 72% (N = 260) of Ensembl geneIDs mapped to identical entries in Galgal4 e73. An additional 30 gene names present in e70, but with different geneIDs, were also mapped to the e73 release. Nineteen gene names that were present in galgal3 e70 were no longer identifiable in the galgal4 e73 release. Possible explanations for their absence include annotation changes, elimination/archival of “novel” genes, and changes to the reference sequence and/or assembly. Overall, 80% (N = 290) of the differentially expressed genes mapping to the e70 release were present in the galgal4 e73 release. Of the 1201 differentially expressed transcripts, 48% (N = 574) were down-regulated in the vulnerable embryos. The four most significant KEGG pathway enrichments in the remapped data set were Ribosome (36 genes, *P* = 1.3 x 10^−17^), Oxidative Phosphorylation (31 genes, *P* = 6.5 x 10^−07^), Cardiac Muscle Contraction (14 genes, *P* = 7.7 x 10^−03^), and Spliceosome (20 genes, *P* = 2.9 x 10^−02^). These enriched KEGG pathways were identical to those identified in our previous study (S3 Table). The GO term clustering also remained unchanged by the remapping.

The intersection of the alcohol-treated (3422 genes) and experimentally-naïve alcohol-vulnerable (1201 genes) data sets contained 525 genes (S4 Table). KEGG pathway analysis of the 525 genes again revealed that the most prevalent enrichments were for Ribosome (36 genes, *P* = 6.8 x 10^−30^), Oxidative Phosphorylation (25 genes, *P* = 1.8 x 10^−10^), Spliceosome (7 genes, *P* = 8.6 x 10^−01^), and Cardiac Muscle Contraction (10 genes, *P* = 2.5 x 10^−03^) (Table 3). Within this common set of 525 genes, we then identified the subset of genes that shared the same directional change in transcriptional abundance in vulnerable/resistant and alcohol-treated neural folds (Table 4). We hypothesized that this subset may contain candidate genes that, when exposed to alcohol, exhibit transcriptional changes in a manner even more pronounced than that observed in vulnerable progenitor cells. The number of overlapping genes in these two sets that concordantly increased or decreased transcript expression was 26 and 243, respectively (S5 Table). The remaining 256 genes had discordant transcript expression.

**Table 3:** Comparison of KEGG Pathway Enrichments and Gene Overlap between Alcohol-Exposed and Experimentally-Naïve Alcohol-Vulnerable Gene Sets.

KEGG pathway	Alcohol Exposed	BH-FDR Significance*	Alcohol Vulnerable	BH-FDR Significance	Gene Overlap	BH-FDR Significance
Ribosome	53 (67)	1.2 x 10^−17^	36	1.3 x 10^−17^	36	6.8 x 10^−30^
Oxidative Phosphorylation	60	4.8 x 10^−12^	31	6.5 x 10^−07^	25	1.8 x 10^−10^
RNA Polymerase	15	2.2 x 10^−03^	4	9.7 x 10^−01^	4	5.9 x 10^−01^
Spliceosome	39	2.6 x 10^−02^	20	1.0 x 10^−03^	7	8.6 x 10^−01^
Cardiac Muscle Contraction	20	2.2 x 10^−01^	14	2.0 x 10^−04^	10	2.5 x 10^−03^

* Benjamin-Hochberg False Discovery Rate

**Table 4:** Differentially-Expressed Genes Having the Same Directional Change in Alcohol-Exposed and Experimentally-Naïve Alcohol-Vulnerable Cranial Neural Folds, Grouped by Function.

	Decreased Expression		Increased Expression	
General Function	# Genes	Gene Names	# Genes	Gene Names
All Overlapping Genes	**243**	**See S4 Table**	**26**	**See S4 Table**
Ribosome	29	RPL7, RPL10A, RPL12, RPL17L, RPL18A, RPL19, RPL21, RPL22, RPL23, RPL23A, RPL24, RPL26, RPL27, RPL29, RPL35, RPL35A, RPL36, RPL37, RPL37A, RPL38, RPL39, RPLP0, RPS11, RPS4, RPS8, RPS17, RPS23, RPL11/TCEB3, RPL30	0	
Ribosome-Related Genes	18	AURKAIP1, DOHH, EBNA1BP2, EIF1, EIF1AY, EIF3D, EIF3K, EIF4E2, GNB2L1, MRPL9, MRPS34, MRTO4, NOL12, NOP56, POLR1D, POLR3K, TMA7		
Oxidative Phosphorylation	17	ATP5A1W, ATP5B, ATP5G1, COX8A, GGA.42010, MT-CO1, MT-CYB, ND4L, NDUFB6, NDUFB8, NDUFB9, NDUFS5, NDUFS7, NDUFS8, SDHC, UQCR10, UQCR11		
Oxidative Phosphorylation-Related Genes	5	ALDOC, GAPDH, IDH2, NFU1, PGAM1		
Cardiac Muscle Contraction	12	ACTC1, DES, GATA6, MYL2, NKX2-5, PNKD, POPDC2, RBM24, TNNT2, TPM4, TRIM55, TTN	2	MTM1, TEAD1
Spliceosome	17	ALYREF, ARL6IP4, BUD31, HNRNPA0, HNRNPA3, HNRNPAB, HNRNPD, HNRNPH3, LSM1, LSMD1, LUC7L2, MAGOH, NONO, NSRP1, SNRPD3, SNRPF, YBX1		
Developmental	9	CHURC1, CRABP2, DKK-1, GGMOXR1, GSK3B, HOXA1, PYGO2, TBX5, WNT4		
DNA Methylation	2	DNMT3A, HDAC8		
Actin Turnover	9	ACTB, ACTG1, CAPG, CAPZB, DBN1, DSTN, SH3BGRL3, TAGLN	1	TNS3
Microtubules	4	DYNLRB1, TUBG1, TUBB2B, MID1		
N-Glycan Synthesis	5	MPI, CALR, PFDN2, SEC13, MVD	1	TNS3
RNA Pol III	7	BTF3, C1QBP, PAF1, POLR2I, SUPT5H, TCEA2, TCEB3,		

### Alcohol-Responsive KEGG Pathways

The high concordance in several gene sets between alcohol-treated and experimentally-naïve alcohol-vulnerable cranial neural folds suggested that these gene clusters might inform mechanisms of alcohol’s action. Based on the analysis of alcohol-treated gene set, we identified five statistically significant KEGG pathways.

#### Ribosomal Proteins

The most significantly overrepresented KEGG pathway in alcohol-treated cells contained 53 genes (*P* = 1.2 x 10^−17^) that encoded small (RPS) and large (RPL) nuclear ribosomal proteins. Because gene annotation in David is not updated regularly, we also searched for terms in the Ensembl gene description matching the specific KEGG pathway (e.g. ribosome, spliceosome, oxidative phosphorylation). We also cross-referenced Ensembl geneIDs to the gene/product names listed in the Gene Ontogeny (GO) database for similar matches, and this identified twelve additional genes encoding RPS and RPL. Furthermore, the annotation for the gene names RPL11 and RPL9 changed to TCEB3 (ENSGALG00000003971) and GGA.41946 (ENSGALG00000000150) respectively. Adding these two genes to the RPL/S gene set gave a grand total of 67 differentially expressed ribosomal protein genes. With the sole exceptions of two kinases, RPS6KA3 (ENSGALG00000016406) and RPS6KA6 (ENSGALG00000007097), all the differentially expressed transcripts had reduced expression in response to alcohol treatment. In addition to reducing transcripts encoding 67 nuclear ribosomal proteins, alcohol treatment also reduced expression of 35 mitochondrial ribosomal proteins. Alcohol’s impact extended to other components in protein translation. This included fifteen aminoacyl-tRNA synthases and two peptidyl-tRNA hydrolases, which provide and recycle charged tRNAs for protein synthesis, and all these had reduced expression.

Comparison of the Ribosome gene set between alcohol-treated (67 genes) and experimentally-naïve alcohol-vulnerable cranial neural folds (36 genes) confirmed the presence of 36 ribosome-associated proteins shared in both gene sets (Table 3). Within this common gene set, 28 shared the same directional change in both alcohol-treated and alcohol-vulnerable cells as compared with their respective controls, and all 28 were decreased. We discovered an additional 18 genes from the overlapping set that were ribosome-related targets, and these included several mitochondrial ribosome proteins, eukaryotic translation initiation factors, and proteins that process rRNA (Table 4). The magnitude of differential expression was consistently greater in alcohol-treated than for alcohol-vulnerable comparisons.

#### Oxidative Phosphorylation

The second most significantly over-represented KEGG pathway in alcohol-treated cells was Oxidative Phosphorylation (*P* = 4.8 x 10^−12^), which contained a cluster of 60 gene entries (Table 3). Of these, nearly all (57) exhibited decreased transcript levels following alcohol challenge, the exceptions being ATP6V1G3 and ATP6V1A, which contribute to V-type ATPase, and the deubiquitinating enzyme ATXN3. Of these 60 genes, 25 also mapped to the experimentally-naïve alcohol-vulnerable gene set and 17 shared the same directional change, all repressed in both alcohol-treated and alcohol-vulnerable cells as compared with their respective controls (Table 4). This intersecting set included genes encoding components of the electron transport chain including the NADH dehydrogenase (ND4L, NDUFB6, NDUFB8, NDUFB9, NDUFS5, NDUFS8, GGA.42010/NDUFV1), ubiquinone-cytochrome C oxidoreductase (MT-CYB, UQCR10, UQCR11), cytochrome oxidase (MT-CO1, COX8A), and F_1_F_0_ATP synthase (ATP5A1W, ATP5B, ATP5G1). Several genes within glycolysis, the TCA cycle, and iron-sulfur cluster biogenesis were also repressed in both cell populations.

#### RNA Polymerase

The KEGG pathway RNA Polymerase was also significantly over-represented with 15 differentially expressed genes in response to alcohol exposure and all but one (PolR1A) were repressed by alcohol (Table 3). Of these, 7 comprise RNA polymerase II, which synthesizes mRNA, and 7 comprise RNA polymerases I and III, responsible for rRNA and tRNA synthesis for use in protein translation (Table 4). Four genes involved in RNA Polymerase were also differentially expressed in alcohol-vulnerable cells, but this was not a sufficient quantity to achieve KEGG pathway significance. Additional genes in the shared data set contribute to the regulation and activity of Pol II.

#### Spliceosome

Of the 39 differentially expressed spliceosome pathway genes present in the alcohol-exposed cells, all but two (AQR, HSP70) had reduced expression in response to alcohol exposure (Tables 3 and 4). Statistically enriched for this pathway (*P* = 2.6 x 10^−2^), 7 spliceosome genes were common to both alcohol-vulnerable and alcohol-treated cells, and of these, peptidylprolyl isomerase H (Cyclophilin H, PPIH) and CCDC12 were reduced in both data sets.

#### Cardiac muscle contraction

Our previous analysis of experimentally-naïve alcohol-vulnerable cells identified the cardiac muscle contraction KEGG pathway as differentially represented [20]. This finding was replicated in the galgal4 remapped gene analysis (Table 3). Although this pathway did not have statistically significant altered representation in response to alcohol (20 genes, *P* = 2.2 x 10^−1^), 14 genes in this pathway overlapped between the two comparisons, omitting those that already were listed in the oxidative phosphorylation pathway. These 14 genes encoded multiple proteins within the contractile apparatus (troponin, α-cardiac actin, titin, desmin, tropomyosin) and transcription factors critical for cardiac specification (NKX2.5, GATA-6, SRF) and differentiation (MEF2C), and most were reduced in response to alcohol, whereas the regulatory effectors TEF1 (TEAD1) and myotubularin-1 (MTM1) were increased.

### Validation of Ribosomal Gene Candidates for Alcohol Vulnerability

We hypothesized that gene transcripts that shared common expression differences between alcohol-vulnerable and alcohol-exposed cells may be candidate genes contributing to alcohol sensitivity. Using an approach established by Swartz et al. [17], we asked if repression of a candidate gene would increase vulnerability to alcohol-induced facial deficits and cellular apoptosis as seen in FASD models. We tested candidate genes in zebrafish, which is amenable to genetic manipulation, has proven utility to evaluate alcohol-sensitive genetic loci [8,17], and undergoes the same calcium/CaMKII-dependent apoptosis as the chick embryo at comparable developmental stages [12]. We focused on ribosomal protein genes because they were the most significantly overrepresented KEGG pathway (Table 2), and they contribute to neural crest development [34]. We selected three ribosomal proteins having significantly reduced expression in alcohol-exposed cells and that are linked to facial deficits in Diamond-Blackfan anemia (RPL5; *zrpl5a*, RPL11/TCEB3; *zrpl11*) [34] or are known to affect facial development (RPS3A; *zrps3a*) [27]; the *zrpl3a* and *zrpl11* morphants recapitulate their transgenic insertional or CRISPR mutation [35–37]. To test for gene-alcohol interactions, we employed concentrations of morpholinos and alcohol that, when administered individually, had no or modest effects on craniofacial development.

**Fig 1:**
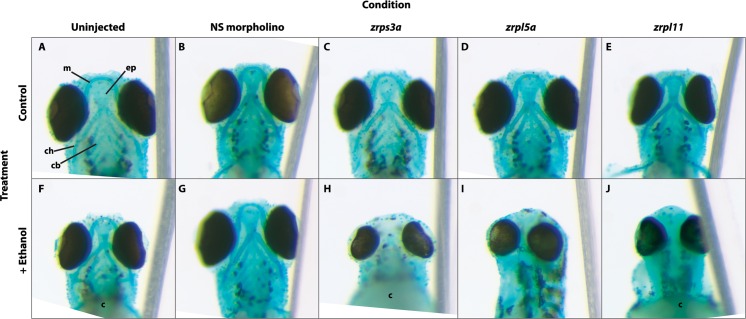
*zrps3a*, *zrpl5a* or *zrpl11* knockdown heightens vulnerability to alcohol-induced craniofacial deficits in zebrafish embryos. (A-E) Untreated embryos (A) had normal development of cranial cartilage elements, and these were largely unaffected by treatment with a nonsense morpholino (B) or morpholinos directed against *zrps3a* (C), *zrpl5a* (D), or *zrpl11* (E). (F-J) Alcohol treatment modestly reduced the size and shape of cranial cartilage elements in otherwise normal embryos. Treatment with a nonsense morpholino (G) did not further worsen cranial development. However, the combination of alcohol with morpholino directed against *zrps3a* (H), *zrpl5a* (I), or *zrpl11* (J) resulted in ablation of many cranial cartilage elements and reduced ocular size. Embryos are either 4 dpf (A-D, F-I) or 3 dpf (E, J). All views are ventral with equivalent magnification. Abbreviations used: c, cardiac edema; cb, ceratobranchial; ch, ceratohyal; ep, ethmoid plate; m, Meckel’s cartilage.

At four dpf, control zebrafish exhibited normally developed cartilaginous head skeletons that included the Meckel’s, basihyal, hyosymplectic, and ceratobranchial cartilages of the lower jaw, and the upper jaw cartilages of the ethmoid plate and trabecula (Fig 1A). Alcohol exposure reduced the embryo’s overall size and caused modest hypotelorism, cranial skeletal asymmetries, and ocular size (Fig 1F). Mandibular cartilage elements were also smaller, and the ethmoid plate was narrowed, compressed, and shortened, observations consistent with prior work [17,30,31]. Because morpholinos can have off-target effects, we tested at equivalent concentrations a negative control morpholino, and this did not adversely affect craniofacial development of nonsense (NS, Fig 1B) or alcohol-treated embryos (Fig 1G). Morpholino repression of *zrps3a* (Fig 1C) did not induce appreciable anomalies of the cartilaginous head skeleton, whereas the addition of alcohol to the *zrps3a* morphants produced profound facial dysmorphologies that ablated most cartilaginous elements of the mandible and neurocranium. Cardiac edema and reduced ocular size were also frequent (Fig 1H). Similar results were obtained with morpholino *zrpl5a* (Fig 1D and 1I), which is one of two gene duplications encoding the paralog of *rpl5*. Again, the control low-dose embryonic injection of *zrpl5a* morpholino did not affect cranial morphogenesis (Fig 1D). When treated with alcohol, these same embryos had ablated cranial elements including the mandible and neurocranium, and marked hypotelorism (Fig 1I). The *zrpl11* morphants were slightly smaller and had normal cartilaginous head skeleton (Fig 1E). Upon addition of alcohol, the most pronounced morphological change was a considerably foreshortened Meckel’s cartilage with less curvature (Fig 1J), hypotelorism, and pronounced cardiac edema. Overall, alcohol administration caused substantial craniofacial cartilage reductions and losses in the ribosomal protein hypomorphs (Fig 1H–1J) as compared with the untreated hypomorphs (Fig 1C–1E), or as compared with alcohol treatment of wild-type or nonsense-treated embryos (Fig 1F and 1G).

**Fig 2:**
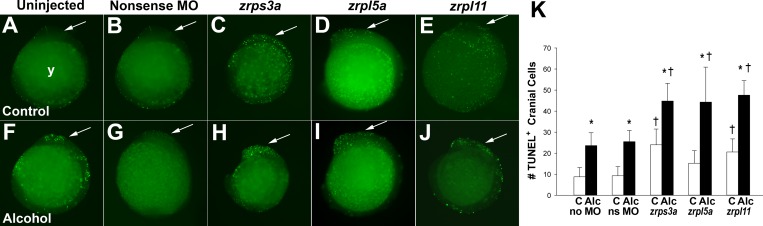
*zrps3a*, *zrpl5a*, or *zrpl11* Knockdown Heightens Vulnerability to Alcohol-Induced Apoptosis in 12 hpf Zebrafish Embryos. (A-E) Untreated embryos (A) had few TUNEL+ cells in the cranial region (arrow). Treatment with a nonsense morpholino (B) caused a modest increase in TUNEL^+^ cells within the cranial region (arrow), as did morpholinos directed against *zrps3a* (C), *zrpl5a* (D), or *zrpl11* (E). (F-J) Alcohol treatment (F) caused appreciable apoptosis within both cranial and somatic regions. The addition of nonsense morpholino treatment (G) did not further increase TUNEL^+^ cell numbers in alcohol-treated embryos. However, the combination of alcohol with morpholino directed against *zrps3a* (H), *zrpl5a* (I), or *zrpl11* (J) resulted in higher levels of apoptosis within the cranial region as compared with embryos that received the same morpholino and no alcohol (C-E), or alcohol and no morpholino (F). All views are lateral at equivalent magnification, with rostral at the top and the embryo ‘looking’ left. Arrow indicates the cranial region. (K) Enumeration of TUNEL^+^ cranial cells in alcohol- and morpholino-treated embryos. Values are mean ± S.D. with 10–12 embryos per treatment. ^*^ Alcohol group differs from no-alcohol control within a treatment at *P*<0.05. ^†^ Morpholino-treated differs from its irrelevant-morpholino control at *P*<0.05. Abbreviation used: Alc, alcohol; C, control; MO, morpholino; y, yolk.

The craniofacial malformations of the alcohol-exposed, ribosomal protein hypomorphs were accompanied by an increased occurrence of cellular apoptosis. At 12 hpf (4–6 somites), untreated zebrafish embryos exhibited only a few TUNEL+ apoptotic cells in the cranial (Fig 2A, yellow arrow; Fig 2K) and caudal regions (clockwise from arrow). Addition of low-dose nonsense morpholino increased slightly the number of TUNEL+ cells (Fig 2B). Morpholino concentrations not affecting facial outcome (Fig 1C–1E) caused a modest rise in apoptosis for *zrps3a* (Fig 2C; *P*<0.05) and *zrpl11* (Fig 2E; *P*<0.05) but not *zrpl5a* (Fig 2D). As expected, alcohol exposure increased the number of TUNEL+ cells within cranial and more caudal regions (arrows, Fig 2F and 2K; *P*<0.05), and the combination of alcohol with *zrps3a*, *zrpl5a*, or *zrpl11* morpholino, greatly expanded the number of TUNEL+ cells within cranial regions, and to a lesser extent more caudally (Fig 2H–2J; *P*<0.05). However, this was not observed with the nonsense morpholino treated embryos (Fig 2G).

## Discussion

Comparative transcriptome profiling has identified pathways and genes that are altered within 6hr of alcohol exposure, providing insights into mechanisms underlying alcohol’s teratogenicity. Pathways of gene clusters having the greatest responsivity included Ribosome, Oxidative Phosphorylation, RNA Polymerase, and Spliceosome. The preponderance of transcripts within those clusters had reduced expression in response to alcohol. These pathways mediate nuclear information flow and comprise a significant portion of the cellular energy budget, particularly for the rapidly proliferating cells that comprise the early embryo.

### Ribosome Biogenesis and Craniofacial Morphogenesis

The significant dysregulation and suppressed transcription within the ribosome protein pathway offers a novel insight into the mechanism underlying neural crest vulnerability and the craniofacial deficits caused by PAE. Alcohol broadly impacted this pathway, with significant reductions in both nuclear and mitochondrial ribosomal proteins, aminoacyl-tRNA synthases and hydrolases, and components of RNA polymerase I and III. Microarray approaches in mouse embryos of comparable stage similarly flagged decreased ribosome transcripts as differentiating the alcohol response, although they were suppressed in the alcohol-vulnerable strain in one study (B6J vs. DBA/J [18]) and in the alcohol-resistant strain in another (B6N vs. B6J [19]); the discrepancy may reflect differences in dose, exposure route, or analytical platforms. The consistent repression of this gene cluster across models implicates ribosomes in modifying alcohol vulnerability. Mechanistic insight is provided by demonstrations that hypomorphs in several ribosome protein pathway participants in zebrafish (*zrps3a*, *zrpl5a*, *zrpl11*, *mars*; [17] and herein) have greater vulnerability to alcohol-induced apoptosis and craniofacial malformations. Genetic loss-of-function in ribosome biogenesis is also causative in several syndromes that feature craniofacial dysmorphology [34]. In Diamond-Blackfan Anemia, loss-of-function in RPL5, L11 or L26, or RPS7, S17, S19, or S26 is associated with Cathie facies, short stature, and upper limb, heart, and urogenital anomalies [38]. Impaired ribosome biogenesis is also causative in the neurocristopathy Treacher-Collins Syndrome [39]. The responsible gene, TCOF1, encodes the nucleolar protein treacle essential for rRNA transcription [40]. Importantly, TCOF1 loss-of-function causes neural crest apoptosis and hypoplasia at the same developmental stages that are vulnerable to alcohol [41]. A similar role for the RNA polymerase subunit POLR1C was recently described [42]. The striking parallels between ribosome dysbiogenesis and alcohol exposure with respect to neural crest survival and facial outcome, and its significant suppression in response to alcohol, propose the ribosome pathway as a candidate for causality in FASD facial dysmorphology.

The loss of ribosome production significantly affects neural crest vulnerability to alcohol. One explanation for this sensitivity is these cells’ exceptional proliferative rate (60%-80%) [43,44], as ribosome biogenesis occupies a substantive fraction of cellular resources and is therefore tightly coordinated with nutrient availability [45]. Insight comes from demonstrations that p53 inactivation or loss-of-function prevents neural crest apoptosis and normalizes facial outcome in animal models of ribosome dysbiogenesis [46–48]. Because of its high energy demand, dysfunction in ribosome biogenesis acts as a signal of nucleolar stress [49,50]. Under such conditions, components of the ribosome complex become limiting and free ribosome proteins exit the nucleolus to bind and inactivate murine double minute-2 (MDM2), an ubiquitinase and repressor of p53. Their interaction stabilizes and activates p53, which then initiates cell cycle arrest and apoptosis. p53 is elevated in alcohol-treated neural crest [51] and small molecule p53 inhibitors prevented these cells’ apoptosis (Flentke, Garic, Berres, and Smith, submitted). The increased cranial apoptosis and skeletal deficits in response to alcohol and hypomorphic ribosome protein content is compatible with such a mechanism.

The mechanism by which alcohol may induce nucleolar stress is unknown. One possibility is that transcriptional losses in electron transport genes contribute to an increased cellular energy deficit. Alcohol suppresses mitochondrial energy generation in diverse cell types, in part through reduced mitochondrial ribogenesis and protein synthesis [52]. The significant repression of transcripts encoding mitochondrial ribosomal proteins and oxidative phosphorylation components is consistent with such a mechanism, and preliminary data suggest that these embryos undergo a sharp reduction in oxygen consumption after exposure to alcohol (Garic, Berres, and Smith, unpublished data). Ribosome biogenesis is additionally linked to cellular energy flux through the actions of mTORC1, which promotes ribosome biogenesis through phosphorylation of RPS6K and RPS6 under anabolic conditions and is suppressed by AMPK when ATP is limiting [53]. Activation of mTOR by L-leucine in *pdgfra* loss-of-function mutants mitigates craniofacial defects in a zebrafish model of FASD (8), implicating suppression of this pathway in alcohol’s mechanism of action. Reduced energy flux through oxidative phosphorylation could be a means to precipitate a loss in ribosome biogenesis and a nucleolar stress condition in response to alcohol exposure.

### Mechanisms of Teratogenesis in PAE

Alcohol impairs multiple events of neural crest development including specification, migration, survival, and differentiation [9]. Alcohol exposure prior to neurulation reduces the prechordal plate [54,55] through its suppression of sonic hedgehog signaling, and thereby reduces both brain size and neural crest cell numbers [55,56]. The midline deficits in the brain and face that partly characterize FAS are linked through this shared developmental origin [57]. The observed reductions in the midline signals SHH and PTCH2, as well as brain segment specification factors such as HOXA1, HOXA2, HOXB5, EN1, DBX1, BMP4, and CRABPI and II, are consistent with this mechanism of alcohol action. Surprisingly, although the alcohol exposure window targeted events of neural crest induction, there were no expression-level changes in genes that govern neural crest induction including SNAI1, SNAI2, FOXD3, WNT6, and WNT1. This suggests that the neural crest reductions that occur in response to alcohol involves mechanisms distinct from fate determination.

Cardiac abnormalities are among the most common birth defects and affect 0.7% of live births [58]. The transcriptional data also offer insights into the mechanisms by which PAE produces heart defects [59]. Although the heart tube was excised prior to analysis, the cranial tissue also includes cells of the secondary heart field, which resides in the lateral plate mesoderm immediately adjacent to the neural folds and forms the distal outflow tract myocardium and smooth muscle of the arterial trunks [60]. At this developmental stage, these early cardiomyocytes begin to differentiate [61]. The reduced expression of numerous contractile proteins suggests that alcohol impaired this differentiation process [61]. This interpretation is supported by significantly reduced expression of regulatory factors that drive formation of the primary and secondary heart fields, including MEF2C, NKX2.5, GATA6, HAND1, TBX5, SRF, and HES1. This may reflect the accompanying reductions in pro-cardiogenic signals such as BMP4, SHH, and WNT3A [61]. Even transient reductions in contractile protein expression may reduce cardiac output and thereby cause looping and valvuloseptal malformations [62]. Because 10.2% of pregnant women in the U.S. report any alcohol use and 3.1% report binge drinking [63], these findings suggest that alcohol abuse may be a greater contributor to cardiac deficits than generally appreciated.

### Conservation of Transcriptional Responses to Alcohol

Many of the observed gene expression changes were also reported in studies of early mouse neurogenesis, suggesting these responses to alcohol are common and conserved. A microarray study of mouse head folds (E8) 3hr after maternal alcohol exposure [19] identified 577 transcriptional changes, of which 143 (24.8%) also had altered representation in our RNA-Seq data set, including genes encoding ribosome proteins, proteasomes, splicing, and energy production. Downing et al. [18] also found significant ribosomal and mRNA splicing enrichments in the comparison of vulnerable and resistant mouse strains 3hr after maternal alcohol exposure at E9.3. Comparisons at the individual gene level were not meaningful due to differences in analytical platforms and the dynamic annotation of these gene sets. Although other functional enrichments were not present in our study, this can be explained by differences in analytical platforms, alcohol dose and timing, model organism, and the incomplete gene annotation. The shared detection of ribosome and splicing enrichments in these models do suggest conserved responses to alcohol during early organogenesis.

### Alcohol-Responsive Genes Predict Genes that Confer Alcohol Vulnerability

Gene clusters having the greatest altered representation between control and alcohol-treated cells–ribosomal proteins, oxidative phosphorylation, and spliceosome–were the same gene clusters having the greatest differential representation between experimentally-naïve alcohol-vulnerable and alcohol-resistant cells [20]. These same gene clusters also emerged in the original mapping of differential vulnerability and thus the remapping did not alter that study’s central conclusions [20]; rather, it further enhanced its data quality. In nearly all differentially expressed genes encoding ribosomes and oxidative phosphorylation, expression was reduced in response to alcohol, thus suggesting that alcohol vulnerability may have been conferred by reduced expression of these genes. Our functional assays in zebrafish support this hypothesis with respect to ribosomal proteins. Reductions in three ribosomal protein genes sensitized the embryos to alcohol-induced apoptosis, facial malformations, and cardiac irregularities. Although the use of morpholinos for gene knockdown is a limitation of this work due to a potential for off-target effects [29], we observed this gene-environment interaction at hypomorphic morpholino concentrations that in themselves caused only modest facial alterations [27,35–37]. Identification of the ribosome protein enrichment pathway is also consistent with a recent genetic screen that identified methionine tRNA synthase (MARS) loss-of-function as enhancing alcohol vulnerability [17]. In the current study, alcohol significantly reduced the expression of fifteen aminoacyl tRNA synthases including MARS and more broadly implicates tRNA metabolism in alcohol vulnerability. Transfer RNA metabolism may have a mechanistic role because a ready supply of aminoacyl-tRNA is necessary for ribosome biogenesis and translation activity [64]. Additional alcohol vulnerability candidates held in common with the zebrafish model include FOXI2 (0.763-fold), VANGL1 (1.806-fold), and PDGFRB (1.976-fold) [8,17]; SNPs within PDGFRB and PDGFRA are associated with altered facial outcome in individuals with PAE [8].

In summary, our results highlight this approach’s efficacy to formulate new, mechanistic hypotheses regarding alcohol’s developmental damage. Ribosome biogenesis may be a novel and primary target modulating alcohol’s damage to developing neural crest populations, and its linkage is consistent with the known effects of ribosome dysbiogenesis on this cell population. This work validates that gene-environment interactions contribute to vulnerability in FASD.

## Supporting Information

S1 Fig Quality Analysis for High-Throughput Sequencing of Alcohol-Treated and Alcohol-Untreated Cranial Neural Folds. (A, B) (A) Plot of normalized expression vs. magnitude of dispersion for control and alcohol treatments. Black dots indicate the empirical dispersion. Line of best fit is indicated in red. (B) Plot of normalized expression versus log2 fold-change (mean expression alcohol/control) in transcript abundance. Transcripts with significantly different abundance following alcohol treatment are shown in red.
(PDF)

S2 Fig Gene Ontologies (GO) Having Significant Differential Expression in Alcohol-Exposed Cranial Neural Folds. (A-C) For each ontology, the number of represented gene transcripts for each term is indicated. For each term category, the percentage of transcripts is indicated. (A) Biological Processes. (B) Cellular Component. (C) Molecular Function.
(PDF)

S1 Table List of 3422 Genes Having Differential Expression in Alcohol-Exposed Cranial Neural Folds. Red boxes indicate genes having differential annotation entries.
(XLSX)

S2 Table List of 1201 Differentially-Expressed Transcripts in Experimentally-Naïve, Alcohol-Vulnerable versus Alcohol-Resistant Cranial Neural Foldsas Mapped onto the Galgal4 e73 Assembly. Red boxes indicate genes having different annotation entries. This table represents a reanalysis, using new annotations, of the sequencing data originally described in [18].
(XLSX)

S3 Table Comparison of Differentially Represented KEGG Pathways in Alcohol-Vulnerable Versus Alcohol-Resistant Cranial Neural Folds in Galgal3 (e70) and Galgal4 (e73). Galgal3 e70 data were obtained from Garic et al., 2014 [18]. The two parenthetical values reflect additional genes in the KEGG pathway between the two releases with different geneID or name entries.
(DOCX)

S4 Table List of 525 Differentially Expressed Genes Held in Common between Alcohol-Exposed and Alcohol-Vulnerable Cranial Neural Folds. (XLSX)

S5 Table Expression-Level Comparisons of 525 Differentially Expressed Genes Held in Common between Alcohol-Exposed and Experimentally-Naïve, Alcohol-Vulnerable Cranial Neural Folds. (XLSX)
