# Supplementary material for: Transcriptome Profiling Identifies Ribosome Biogenesis as a Target of Alcohol Teratogenicity and Vulnerability during Early Embryogenesis
Source: PLoS One. 2017 Jan 3;12(1):e0169351. doi: 10.1371/journal.pone.0169351 (PMC5207668; doi:10.1371/journal.pone.0169351)
Supplement: S1 Fig — (A, B) (A) Plot of normalized expression vs. magnitude of dispersion for control and alcohol treatments. Black dots indicate the empirical dispersion. Line of best fit is indicated in red. (B) Plot of normalized expression versus log2 fold-change (mean expression alcohol/control) in transcript abundance. Transcripts with significantly different abundance following alcohol treatment are shown in red. (PDF) [file pone.0169351.s001.pdf]

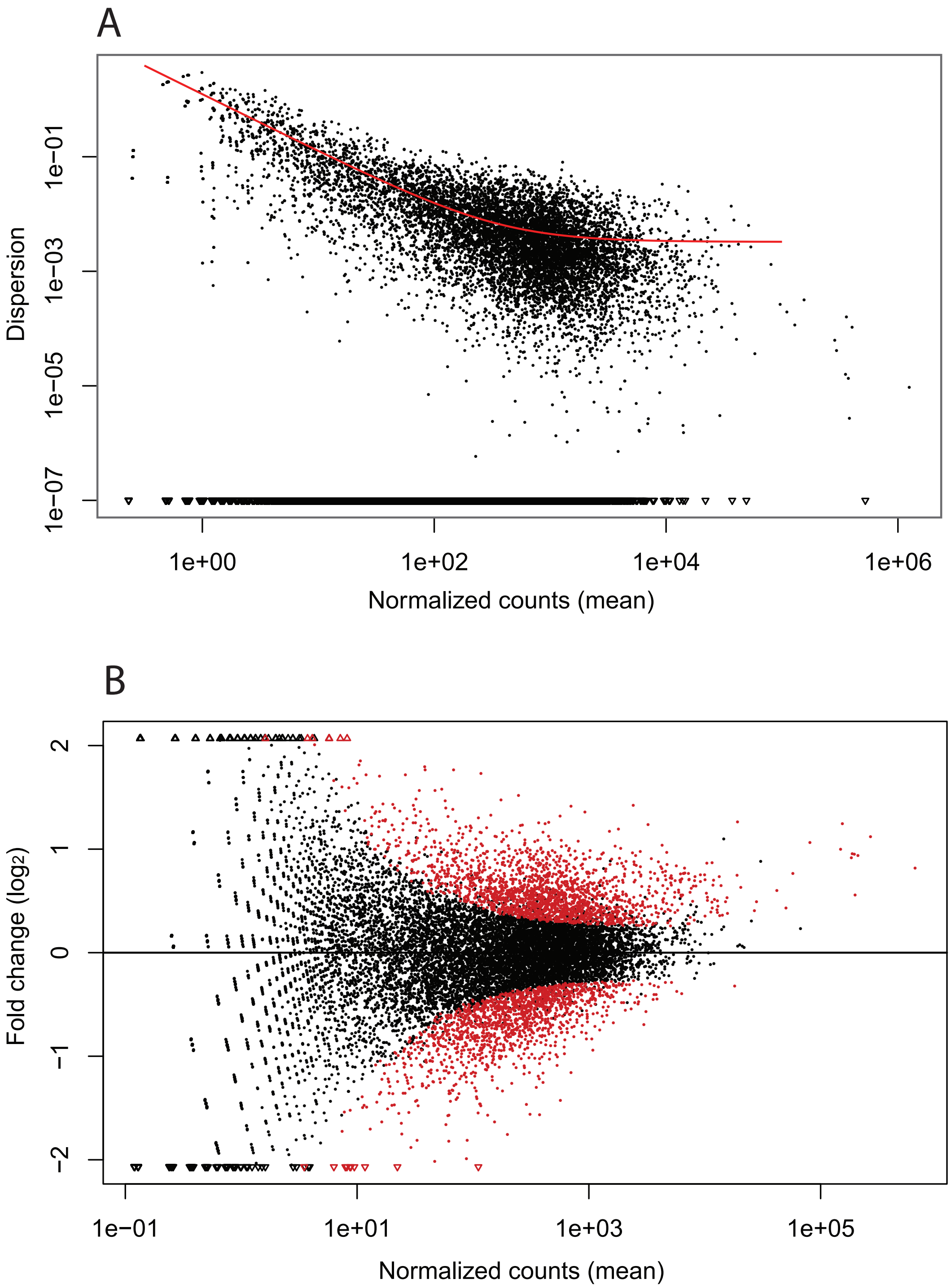

Fig S1. Quality analysis for high-throughput sequencing of alcohol-treated and alcohol-untreated neuroprogenitor cells. (A,B) (A) Plot of normalized expression vs. magnitude of dispersion for control and alcohol treatments. Black dots indicate the empirical dispersion. Line of best fit is indicated in red. (B) Plot of normalized expression versus log2 fold-change (mean expression alcohol/control) in transcript abundance. Transcripts with significantly different abundance following alcohol treatment are shown in red.
