# Supplementary material for: Transcriptome Profiling Identifies Ribosome Biogenesis as a Target of Alcohol Teratogenicity and Vulnerability during Early Embryogenesis
Source: PLoS One. 2017 Jan 3;12(1):e0169351. doi: 10.1371/journal.pone.0169351 (PMC5207668; doi:10.1371/journal.pone.0169351)
Supplement: S2 Fig — (A-C) For each ontology, the number of represented gene transcripts for each term is indicated. For each term category, the percentage of transcripts is indicated. (A) Biological Processes. (B) Cellular Component. (C) Molecular Function. (PDF) [file pone.0169351.s002.pdf]

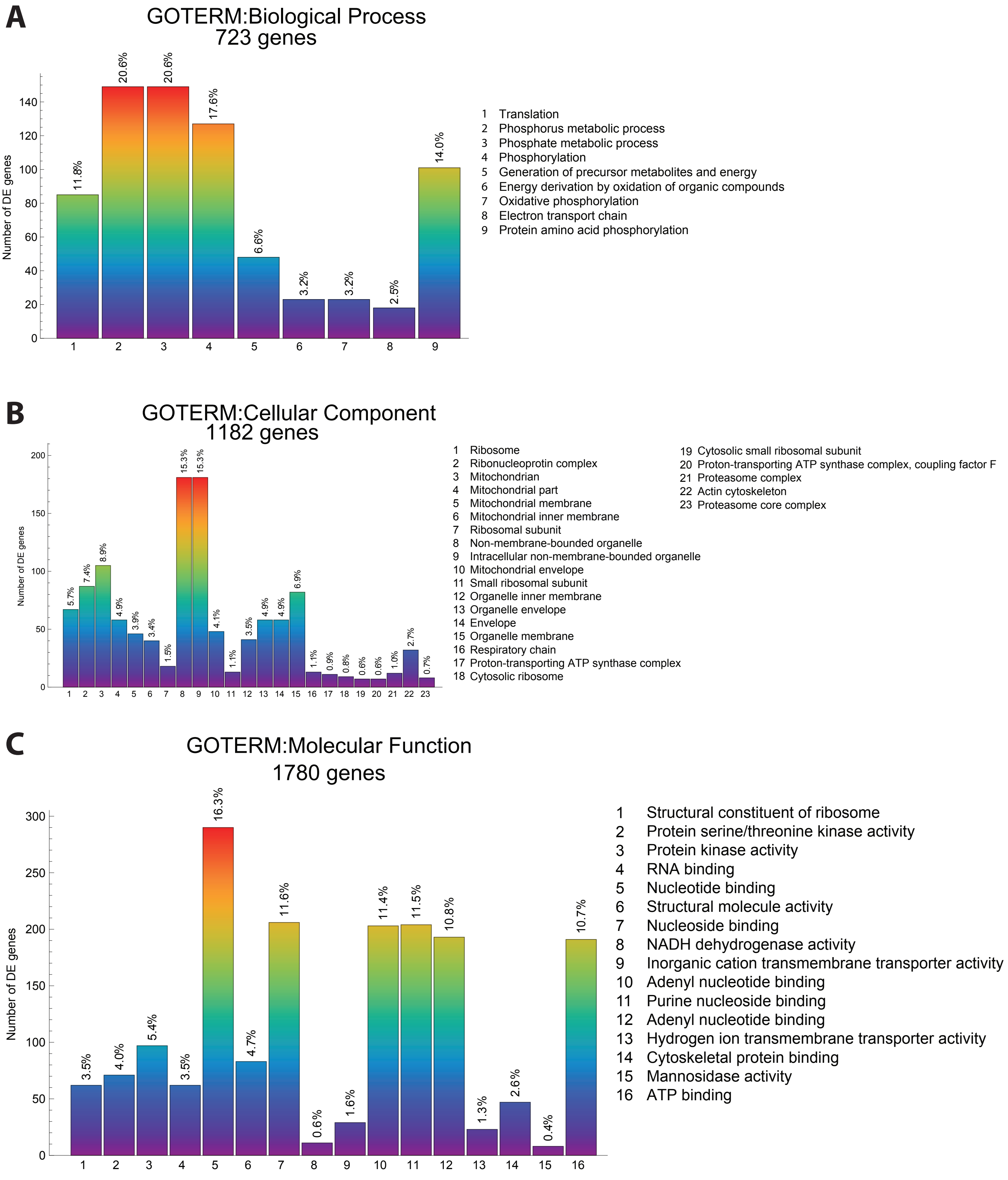

Fig S2. Gene ontologies (GO) having significant differential enrichment in alcohol-exposed neuroprogenitors. (A-C) For each ontology, the number of represented gene transcripts for each term is indicated. For each term category, the percentage of transcripts is indicated. (A) Biological Processes (B) Cellular Component (C) Molecular Function.
