# Supplementary material for: Transcriptome Profiling Identifies Ribosome Biogenesis as a Target of Alcohol Teratogenicity and Vulnerability during Early Embryogenesis
Source: PLoS One. 2017 Jan 3;12(1):e0169351. doi: 10.1371/journal.pone.0169351 (PMC5207668; doi:10.1371/journal.pone.0169351)
Supplement: S3 Table — Galgal3 e70 data were obtained from Garic et al., 2014 [18]. The two parenthetical values reflect additional genes in the KEGG pathway between the two releases with different geneID or name entries. (DOCX) [file pone.0169351.s005.docx]

| KEGG pathway | Galgal3 e70^*^ | BH significance | Gagal4 e73 | BH significance | Overlap in galgal4^**^ | BH significance |
| --- | --- | --- | --- | --- | --- | --- |
| Ribosome | 36 | 6.3 x 10^-31^ | 36 | 1.3 x 10^-17^ | 31 (35) | 1.9 x 10^-26^ |
| Oxidative Phosphorylation | 20 | 8.1 x 10^-07^ | 31 | 6.5 x 10^-07^ | 16 (20) | 4.4 x 10^-05^ |
| Cardiac muscle contraction | 9 | 7.1 x 10^-03^ | 14 | 7.7 x 10^-03^ | 8 | 1.0 x 10^-02^ |
| Spliceosome | 13 | 8.4 x 10^-03^ | 20 | 2.9 x 10^-02^ | 12 | 5.2 x 10^-03^ |

Table S3. Comparison of differentially represented KEGG Pathways in alcohol-vulnerable versus alcohol-resistant neuroprogenitors in Galgal3 (e70) and Galgal4 (e73).

^*^ Galgal3 e70 data were obtained from Garic et al., 2014.

^**^ The two parenthetical values reflect additional genes in the KEGG pathway between the two releases with different geneID or name entries.
